# Is topical or intravenous tranexamic acid preferred in total hip arthroplasty? A randomized, controlled, noninferiority clinical trial

**DOI:** 10.1371/journal.pone.0204551

**Published:** 2018-10-02

**Authors:** Kai-di Zhou, Hong-yi Wang, Yi Wang, Zhi-hong Liu, Chuan He, Jian-min Feng

**Affiliations:** 1 Department of Orthopedics, Ruijin Hospital, Shanghai Jiao Tong University School of Medicine, Shanghai, China; 2 Shanghai Institute of Traumatology and Orthopedics, Shanghai, China; Copenhagen University Hospital, DENMARK

## Abstract

**Purpose:**

The present study aimed to confirm the efficacy and safety of topical and intravenous tranexamic acid (TXA) compared with that of topical placebo and to assess the noninferiority between the two application methods of TXA in patients undergoing unilateral primary total hip arthroplasty.

**Methods:**

Our randomized controlled trial investigated 170 patients with 1:1:1 allocation to two doses of 10-mg/kg intravenous TXA, 3-g topical TXA, and topical placebo of 60-ml physiological saline groups. The primary outcome, total blood loss, was calculated with Nadler and Gross formula. The secondary outcomes included allogeneic blood transfusion requirement, drain blood loss, decreased hemoglobin level. Noninferiority would be established when the upper limit 95% CI is lower than 250 ml of the noninferiority margin for the mean difference of total blood loss between topical and intravenous TXA. Thromboembolic complication incidence was considered as a safety outcome.

**Results:**

The total blood loss of patients administered intravenous (mean±standard deviation, 1125±514 ml) and topical TXA (1211±425 ml) was significantly reduced compared with that of those administered topical placebo (1464±556 ml) (p = 0.0012). Drain blood loss and hemoglobin level reduction in patients administered with TXA were also significantly lower than those in patients administered topical placebo. The mean difference of total blood loss between topical and intravenous TXA is 86 ml (95% CI, −88 to 260 ml). The complications were comparable between patients managed with TXA and patients with topical placebo.

**Conclusion:**

The noninferiority of topical TXA to intravenous TXA can not be concluded. Considering no significant difference was found in all efficacy outcomes between the two administration methods. Any of the two TXA administration methods can be adopted for blood loss prevention in total hip arthroplasty.

## Introduction

Hemorrhage and subsequent anemia are highly prevalent in patients undergoing total hip arthroplasty (THA) [1, 2].

Postoperative anemia will impede physical functioning, delay rehabilitation, and increase mortality [3]. As a result, approximately one-thirds of the patients may require allogeneic blood transfusion. However, allogeneic transfusion is associated with risks for disease transmission, immunosuppression, and transfusion reactions [4]. Postoperative anemia may impede the patients from physical recovery and increase the risk of postoperative infections. Thus, tranexamic acid (TXA) was introduced as an alternative to reduce perioperative blood loss and allogeneic transfusion requirement [5–7].

Surgical trauma causes hyperfibrinolysis, which induces fibrin clot dissolution to sustain bleeding [8]. TXA act as a lysine analog which inhibits hyperfibrinolysis by blocking the interaction of plasminogen with fibrin to prevent the dissolution of the fibrin clot and thereby reduce bleeding [6–10]. TXA was first applied intravenously (ivTXA) in both total knee and hip arthroplasty, with promising results [7, 11, 12]. Some researchers still doubt whether thromboembolic events increased when TXA is administered systemically. After Wong et al. applied TXA in topically (tTXA) in total knee arthroplasty (TKA) with encouraging results, a growing number of studies comparing tTXA without TXA demonstrated that tTXA can significantly reduce blood loss in TKA without increasing the incidence of thromboembolic complications [13, 14]. Recently, several randomized clinical trials comparing ivTXA with tTXA in total knee and hip arthroplasty found no significant difference regarding total blood loss and transfusion requirement [15–18]. In addition, a high-quality prospective study conducted by Gomez et al. indicated that tTXA demonstrated noninferiority compared with ivTXA in reducing blood loss in TKA [19].

The timing to apply TXA is different between total knee and hip arthroplasty (THA). Both ivTXA and tTXA are recommended before tourniquet release at end of TKA surgery. Thus, tTXA can contribute a much higher concentration in the surgical field than ivTXA and result in maximum efficacy[13]. However, in THA, ivTXA is administered before surgery so that ivTXA can suppress fibrinolysis at the early stage of coagulation cascade and fibrinolysis. Therefore, the optimal route in administering TXA in THA remains unclear.

The first goal of the present double-blinded, randomized trial was to confirm the efficacy and safety of ivTXA and tTXA compared with topical placebo. The second objective was to assess if 3-g tTXA was noninferior to two 10 mg/kg doses ivTXA in reducing blood loss in patients undergoing primary unilateral THA with cementless implants.

## Materials and methods

The present study was registered in the public ClinicalTrials.gov registry (NCT02312440) after approval was granted by ethics board of Ruijin Hospital, Shanghai Jiao Tong University School of Medicine. The complete date range for patient recruitment and follow-up was also approved by ethics board of Ruijin Hospital. In addition, the study protocol has also been published [20]. The authors confirm that all ongoing and related trials for this intervention are registered. However, the trial was registered after patient recruitment began because the translation of the protocol delays its releasement. Written informed consent was obtained from the patients or their legal representatives.

### 2.1 Trial design and participants

This randomized controlled trial was conducted to evaluate and compare the efficacy and safety of two different routes of TXA administered in THA with 1:1:1 allocation to topical placebo, ivTXA, and tTXA groups. All adult patients scheduled to undergo primary unilateral THA in our hospital and consented to participate in our study were eligible for inclusion. Exclusion criteria were allergy to TXA; coagulopathy (preoperative platelet count < 150,000/mm3; international normalized ratio (INR) > 1.4; or any indicator of prolonged partial thromboplastin, prothrombin, and thrombin time of >1.4 times the normal.); history of thromboembolic disease, including deep vein thrombosis (DVT), pulmonary embolism (PE), myocardial infarction (MI), and cerebral infarction (CI); taking anticoagulant drugs within a week before surgery; major comorbidities, including severe ischemic heart disease (New York Heart Association Class III or IV), renal dysfunction (glomerular filtration rate < 60), or hepatic dysfunction (glutamic–pyruvic transaminase > 80 or glutamic oxaloacetic transaminase > 80); retinopathy; pregnancy; participated in another clinical trial within a year; and those who completely stay in bed for more than 3 weeks.

### 2.2 Interventions

Patients assigned to the topical placebo group were administered 60 ml 0.9% sodium chloride solution by soaking the hip cavity at least 3 min before being suctioned at the end of surgery. Patients assigned to the ivTXA group were administered 10-mg/kg TXA in 100 ml 0.9% sodium chloride by intravenous infusion approximately 15 min before skin incision as first dose, and a second identical dose was administered 3 h after the first dose. Patients assigned to the tTXA group were administered 60 ml 0.9% sodium chloride solution containing 3-g TXA by soaking the hip cavity for at least 3 min before being suctioned at the end of surgery. The joint mobility was tested and the X-ray plate was taken after the last implant was finished for every patient. These steps may take 4–5 min. We can finish the hip cavity soaking without prolonging operation time. The dosage of ivTXA and tTXA was based on previous efficacy studies that confirm that two doses of 10 mg/kg to 20 mg/kg ivTXA or 2 g to 3 g tTXA can significantly reduce transfusion rate and total blood loss in joint replacement surgery [21–23].

The perioperative management for the participants in the present study was not different from that for the other patients undergoing THA at our institution. We asked all patients to discontinue anticoagulant drugs at least 7 days before the surgery. All surgeries were performed by four senior surgeons, with the anterolateral approach and cementless prosthesis under controlled hypotension and general anesthesia. An intra-articular drain tube connected to a vacuum bottle was placed in all patients. The drain tube was clamped for 2 h after the surgery and removed on the morning of postoperative day 2. All patients orally took 10-mg rivaroxaban tablets for anticoagulation for 15 days from postoperative day 1. Cephalosporin was used to prevent infection, and clindamycin was used when patients were allergic to cephalosporin. No patients were administered erythropoietin or iron treatment in the perioperative period.

## Randomization and blinding

Computer-generated randomization table with block sizes of 9 was performed to randomize patients a day before the surgery based on their admission sequence. Independent pharmacists prepared the study medication and topical placebo after an investigator provided the intraoperative prescription based on the patient's assignments. In the operation room, a scrub nurse provided a 60-ml solution with or without TXA to surgeons, whereas ivTXA solution was provided to anesthesiologists to administer based on the prescription. Patients, surgeons, and nurses participating in treatment and evaluation were blinded to the group allocation throughout the study period.

### Outcome measures

The primary outcome regarding efficacy is the total blood loss, as calculated with the formula described by Nadler and Gross [24, 25]. One unit of PBRC transfused to the patients is calculated as 200 ml blood loss during the calculation of total blood loss with the Nadler & Gross formula; one unit of packed red blood cell is deemed containing 24 g hemoglobin in the calculation of postoperative hemoglobin loss. Secondary outcome includes drain blood loss and transfusion requirement. Drain blood loss was recorded by a nurse at approximately 36 h postoperative. The demand of intraoperative transfusion is decided by surgeons. Postoperative blood transfusion was required if the patient’s hemoglobin (Hb) level < 80 g/l or < 100 g/l with anemic symptom complications. Other outcomes include intraoperative blood loss, amount of human serum albumin used during hospitalization, coagulation indicator (activated partial thromboplastin time [APTT], thrombin time [TT], prothrombin time [PT], and INR).

Thromboembolic complications were monitored and recorded as a safety outcome. Vascular ultrasound Doppler examination was performed in patients with suspected DVT. The last follow-up was scheduled at postoperative week 6 in our hospital. During the follow-up, the patients can directly report their discomfort to the researchers.

### Sample size and statistical analysis

We hypothesized that tTXA is not inferior to ivTXA. Based on previous studies, ivTXA can reduce about 400 ml blood loss in THA and we deemed that a meaningful difference for the reduction of total blood loss between TXA and placebo is 150 ml [26–28]. Therefor, the noninferiority margin was set at 250 ml which is also the largest clinically difference of tTXA we can accept when compared with ivTXA in reducing blood loss.

The significance level (alpha) of the test is 0.025. The standard deviations were set at 442.2 ml and 439.6 ml in the tTXA and ivTXA groups, respectively, based on a previous study that compared tTXA with ivTXA on estimated blood loss in patients undergoing TKA [19]. Noninferiority would be established when the upper limit 95% CI is lower than 250 ml of the noninferiority margin for the mean difference of total blood loss between tTXA and ivTXA. After a 15% drop-out rate was considered, the patients required for each group was 58 to achieve 80% power to detect noninferiority using a one-sided, two-sample t-test. The sample size was calculated by using PASS 11.0 software. SAS 8 was used for statistical analyses. The normally distributed continuous variables were tested by using one-way analysis of variance (ANOVA), and pairwise variable was compared with Bonferroni correction. Categorical variables were analyzed with the chi-square test. A p-value < 0.05 was considered statistically significant.

## Results

**Fig 1:**
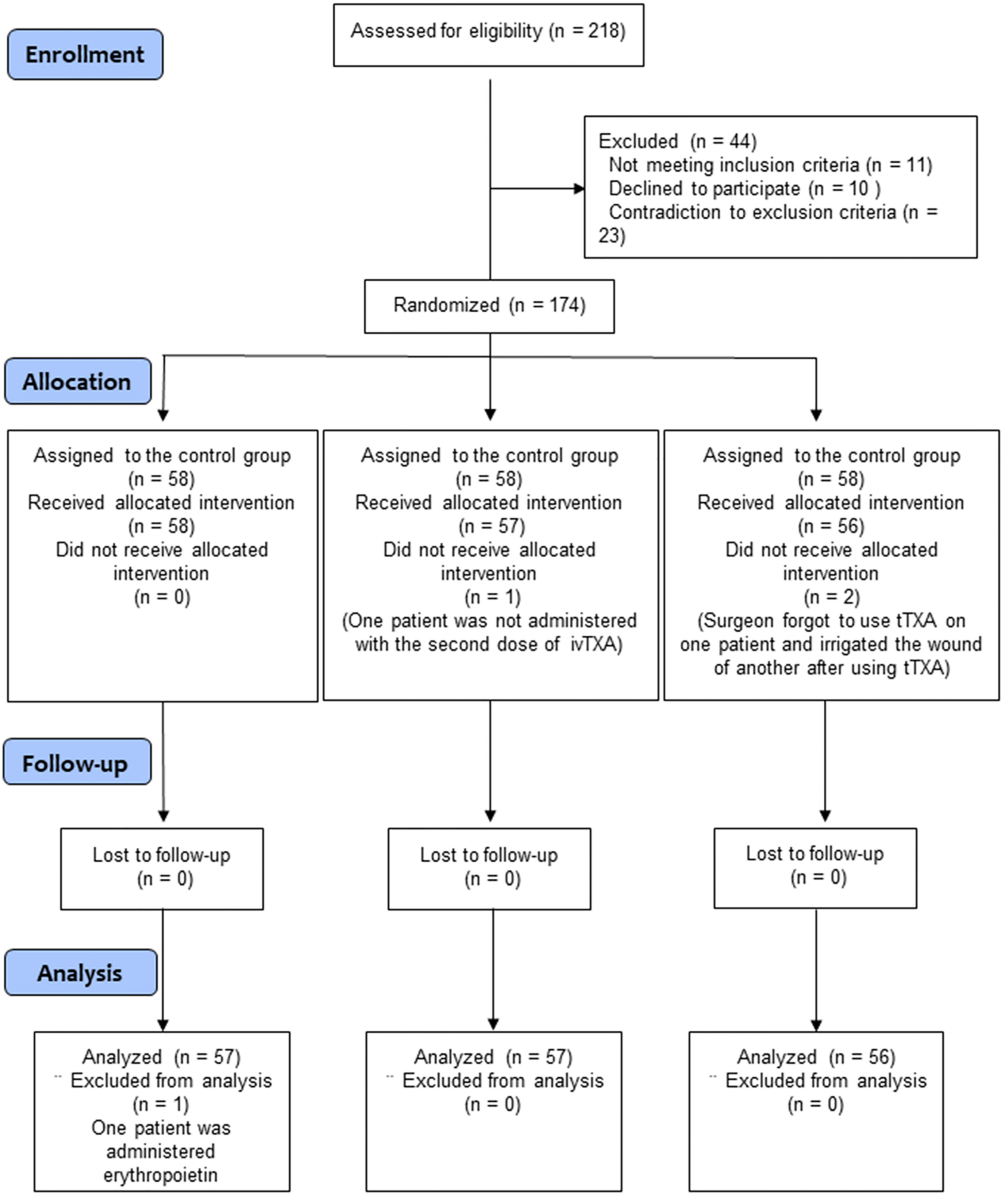
CONSORT diagram for this clinical trial.

From July 2014 to May 2015, 218 patients were scheduled for primary unilateral THA at our department (Fig 1). Based on the inclusion/exclusion criteria, 44 patients were excluded, and 174 were randomized to three cohorts. Of the 174 randomized patients, 170 completed the study and entered in statistical analysis. Fig 1 shows the reasons for excluding four randomized patients. No patient was lost or excluded during the follow-up.

The base-line characteristics included sex, age, weight, height, body mass index, American Society of Anesthesiologists (ASA) status, preoperative laboratory values (Hb, hematocrit, APTT, PT, and INR), or surgical characteristics (surgical duration and disease composition) are showed in Table 1.

**Table 1:** Baseline characteristics of patients in the three groups.

	Placebo group	ivTXA group	tTXA group
Patients	57	57	56
Age (years)	65.3±11.2	63.2±10.0	65.8±9.4
Sex (F/M)	43/14	45/12	39/17
Height (m)	1.6±0.1	1.60±0.1	1.6±0.1
Weight (kg)	61±11.5	60.6±9.7	61±9.0
Body mass index (kg/m)	23.5±3.8	23.7±3.2	23.2±3.1
ASA score			
I	5	7	3
II	48	41	48
III	4	9	5
Preoperative laboratory values			
Preoperative Hb (g/l)	128.2±12.6	127.4±14	129.6±12.8
Preoperative Hct (dl/dl)	0.38±0.04	0.38±0.04	0.39±0.04
PT (s)	10.8±0.6	10.7±0.7	10.7±0.7
APTT (s)	29.3±3.1	28.7±3.4	28.4±3.2
INR (s)	0.92±0.057	0.90±0.095	0.91±0.056
TT(s)	17.4±3.8	18.3±±0.82	17.8±4.0
Surgery characteristics			
Operative time (min)	90.4±26.5	93.9±28.3	91.0±24.1
Femoral neck fracture	15	8	8
Femur head necrosis	20	9	9
Osteoarthritis	10	16	20
Developmental dysplasia of hip	12	24	19

Note: Data are shown as mean±SD.

**Fig 2:**
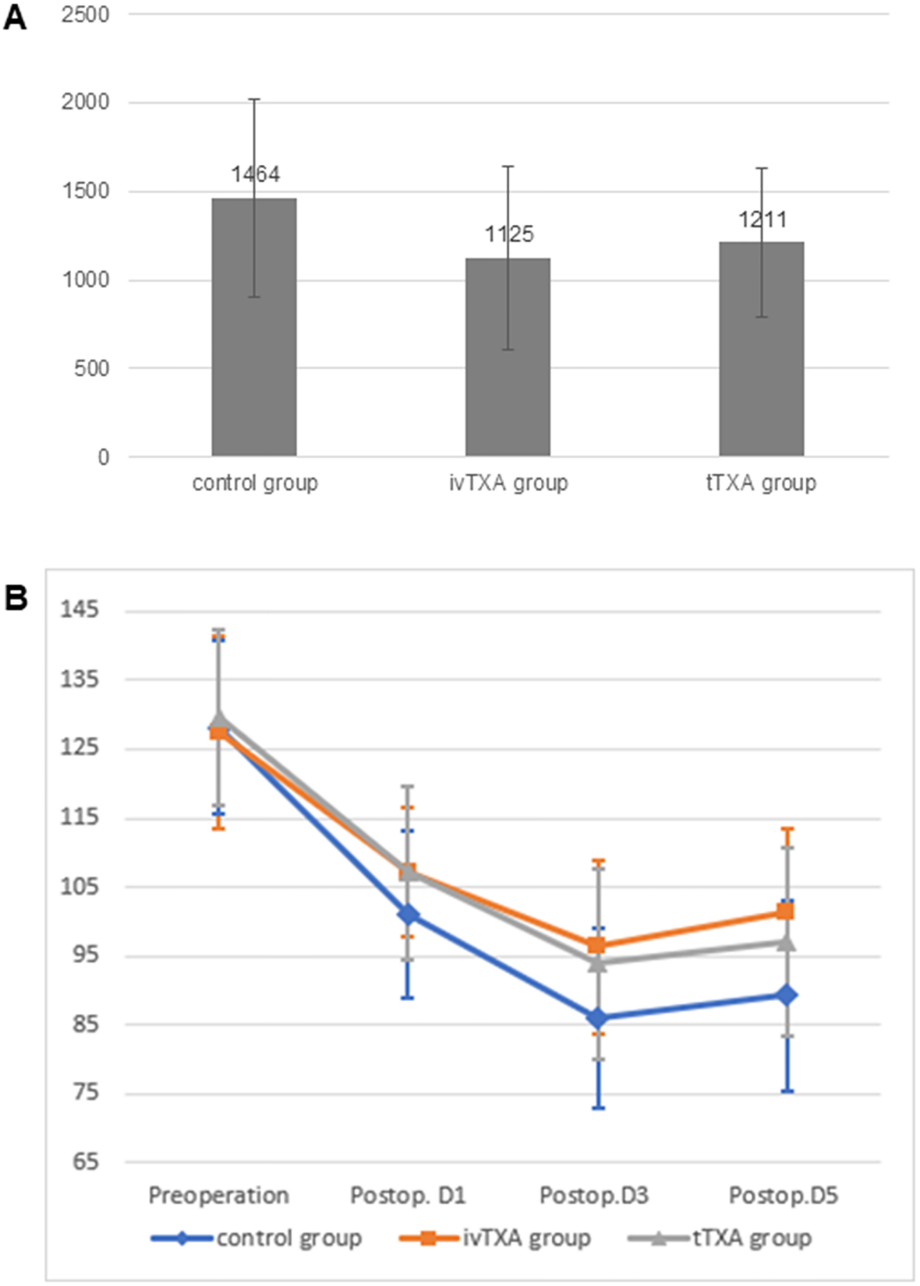
Total blood loss and hemoglobin level. (A) Total blood loss in ml (and 95% CI) in each group. (B) Hemoglobin level in g/l (mean±SD) based on the time in each treatment group.

**Fig 3:**
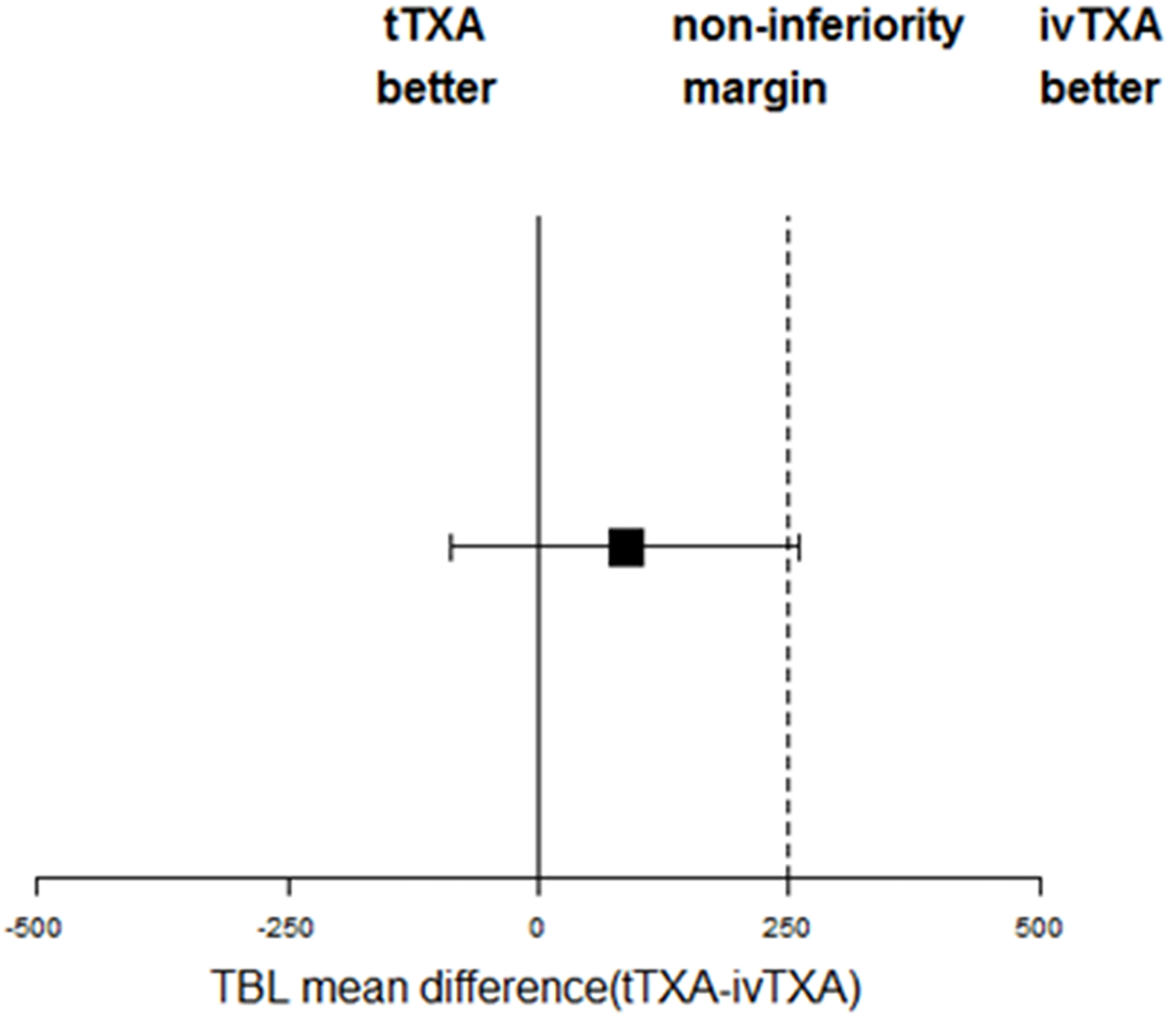
The mean difference in ml (with 95% CI) of total blood loss in patients administered with tTXA compared with total blood loss in patients administered with ivTXA. The upper limit of 95% CI for the mean difference is greater than 250 ml of the noninferiority margin.

Comparing the two TXA groups with the control group, Table 2 and Fig 2A show a significant reduction of total blood loss, drain blood loss, and Hb decrease in patients in the TXA groups than those in patients in the control group. However, no significant difference was detected concerning intraoperative blood loss, transfusion requirement, human albumin requirement, and postoperative coagulation indications between the groups. Comparing ivTXA with tTXA, Table 2 and Fig 2 show that patients in the ivTXA group had lesser total blood loss, drain blood loss, Hb decrease, and higher postoperative Hb level than patients in the tTXA group. In contrast, transfusion requirement was lower in the tTXA group than that in the ivTXA group. However, these differences failed to reach statistical significance. As the primary outcome, total blood loss in the ivTXA group was decreased by 86 ml (95% CI, −86 to 266 ml) compared with that in the tTXA group. The hypothesis that 3-g tTXA was noninferior to two doses of 10-mg/kg ivTXA cannot be established (Fig 3).

**Table 2:** Postoperative outcome.

Outcome	Placebo group (G1)	ivTXA group (G2)	p-value ^c^ G1 vs G2	tTXA group (G3)	p-value^d^ G1 vs G3	p-value ^e^ G2 vs G3	p-value ^a^
Total blood loss (ml)	1464±556	1125±514	0.0012	1211±425	.0244	1.0000	0.0012
Drainage output (ml)	301±181	204±169	0.0058	232±132	.0789	1.000	0.006
Transfusion requirement	30 (52.6%)	24 (42.1%)		20 (35.7%)			0.1864 ^b^
Intraop. blood loss(ml)	397±239	402±229		404±213			0.9863
Postoperative Hb level							
Postop. D1(g/l)	101±12.2	107.3±9.4	0.0112	107.1±12.5	0.0156	1.0000	0.0045
Postop.D3 (g/l)	86±13.1	96.3±12.6	0.0002	93.9±13.8	0.0065	1.0000	0.0002
Postop.D5 (g/l)	89.3±13.8	101.3±12.3	<0.0001	97±13.7	0.0109	0.3119	<0.0001
Hb loss from Preop.Hb							
Postop.D1 (g/l)	33.3±12.3	26.5±12.6	0.0109	27.6±11.9	0.0437	1.0000	0.0077
Postop.D3 (g/l)	48.3±14.8	37.0±15.4	0.0009	40.2±13.3	0.0134	1.0000	0.0007
Postop.D5 (g/l)	46.3±14.8	33.7±14.9	<0.0001	38.1±14.1	0.0144	.3976	<0.0001
Albumin requirement	10 (17.5%)	8(14%)		10(17.8%)			0.8304 ^b^
Postop.PT (s)	12±1.3	11.8±0.9		11.7±1.0			0.3004
Postop.APTT (s)	31.7±6.2	30.0±3.7		29.7±4.6			0.1049
INR(s)	1.02±0.1	1.00±0.08		1.00±0.08			0.3324
Postop.TT (s)	18.8±1.5	18.9±1.2		19.1±1.5			0.6047
Postop.FG	2.97±0.75	2.84±0.66		2.67±0.64			0.0971

Note: Data are shown as mean±SD;

^a^: Using ANOVA for comparisons among groups unless otherwise stated.

^b^: using Chi-Square for comparisons among groups.

^c, d, e^: applying Bonferroni correction for pairwise comparisons.

Three episodes of suspicious DVT were recorded. One was in the control group; the other two, in the tTXA group. However, each of the three had a negative Doppler evaluation. No any other episodes of thromboembolic complications were found during follow-up, and all 174 patients had good recovery at postoperative week 6.

## Discussion

We conducted a randomized controlled trial comparing two application routes of TXA with topical placebo. The first hypothesis that either tTXA or ivTXA would significantly reduce blood loss in patients undergoing THA compared with topical placebo has been confirmed. The second hypothesis that tTXA would be noninferior to ivTXA in conserving blood in patients undergoing THA cannot be established. Considering the lesser blood loss and easier to administer before and after the surgery compared with tTXA, we support administering two doses of 10-mg/kg ivTXA in unilateral primary THA as a standardized protocol for blood loss prevention. Considering the upper limit 95% CI of mean difference of total blood loss is only slightly higher than noninferiority margin and no significant differences were found for other efficacy outcomes when tTXA compared with ivTXA. In addition, tTXA can reduce 252 ml total blood loss when compared with topical placebo.

The mechanism of TXA to reduce bleeding is to stabilize fibrin clots sealing the opened vessels [9]. When applied intravenously, TXA rapidly diffuses in the joint fluid and reach the same concentration as that in the serum [29]. When applied topically, TXA directly targets the bleeding site and is barely absorbed into systemic circulation. Although tTXA reached a higher concentration in the surgical field than ivTXA, the time that we applied the first dose of ivTXA was 15 min before the surgery, which was much earlier than tTXA administration. Previous studies indicated that the cascade process of fibrinolytic activation could be well inhibited at its initial phase. TXA should be applied at the early stage of fibrinolytic activation [6, 30, 31]. Thus, the different application timing of TXA might be the reason why tTXA with high concentrations cannot result in a better effect than low-dose ivTXA in patients undergoing THA.

Our findings of reduced total blood loss correspond with those of previous clinical trials and meta-analyses studying TXA administration in patients undergoing THA [32–34]. Meta-analyses including 11 clinical trials demonstrated a mean reduction of 289 ml total blood loss per case. We revealed blood loss reduction of approximately 327 ml in patients administered with ivTXA, and approximately 252 ml with tTXA, respectively, when compared with topical placebo.

Our results of transfusion requirement agreed with several studies but contradicted to more studies and meta-analysis that demonstrated a significant reduction in transfusion requirement when TXA was administered. In the present study, the transfusion requirement was 10.5% lower in the ivTXA group and 16.9% lower in the tTXA group compared with placebo group. However, Chi-squared test results showed that the allogeneic transfusion requirement was not statistically significant among the TXA and placebo groups. Because we use calculated total blood loss as our primary outcome, we did not exclude anemic patients in present study. The preoperative anemic patients are easier to reach blood transfusion pointer even though TXA reduced their blood loss. Thus, as a predictor of transfusion requirement, preoperative anemia may weaken the effect of TXA for reducing transfusion requirement in the present study.

Several high-quality studies have been published comparing the effects and safety between ivTXA and tTXA. Wind et al. retrospectively reviewed 1595 primary THA and found that both ivTXA and tTXA can significantly reduce blood loss, but tTXA failed to significantly reduce the transfusion requirement compared with no TXA[33]. North et al. concluded that calculated blood and Hb losses were lower in the ivTXA group than in the tTXA group [34]. Thus, Wind et al. and North et al. recommended using ivTXA in THA. In contrast, Wei et al. inclined to administer TXA topically. Because Wei et al. found in their study that tTXA carried a comparable hemostatic effect compared with ivTXA [18]. In addition, tTXA seems to be theoretically safe because a small amount of TXA could be absorbed into the systematic circulation [13]. Interestingly, the above studies administered tTXA in different routes. Wind et al. and North et al. soaked the wound before closure for several minutes by TXA solution as we did in the present study. However, Wei et al. divided the 3-g TXA solution into three parts to administer in three steps: after acetabular preparation, after femoral canal breach preparation, and before closure. Such method was first described in the study conducted by Konig et al., who demonstrated topical TXA could significantly reduce blood loss and transfusion requirement in patients undergoing THA [35]. We inferred that their earlier timing to administer tTXA may maximized its effectiveness.

The timing and dosage of ivTXA and tTXA administered in THA are various. Previous studies usually adopt two doses of 10 mg/kg to 20 mg/kg or one loading dose of 1 g to 2 g TXA as their intravenous route. Topical route of TXA often refers to applying 1-g to 3-g TXA solution by soaking or impregnating the hip cavity. In 2016, Yi Z et al combined 15-mg/kg ivTXA with 1-g tTXA as a new method in TXA application in patients undergoing THA and achieved promising results of reducing bleeding [36]. Recently, Sun Y et al conducted a meta-analysis including five studies demonstrated that the combined TXA can reduce more blood loss without increasing the rate of thromboembolic when compared with ivTXA use alone in THA [37]. However, the optimal method and dosage to apply TXA in THA is still an issue worth exploring for future research.

The thromboembolic complications in the present study included three suspicious DVT, which turned out to be negative by Doppler evaluation. In consistent with many previous clinical trials and meta-analysis, the patients who were administered either ivTXA or tTXA did not have an increase in DVT or overall thromboembolic complications compared with those who were not administered TXA [32]. Of note, we excluded patients at thromboembolic risk as many relevant studies did.

Our study has limitations. First, the sample size of the present study was not big enough for detecting the difference of complications with low incidences, such as pulmonary embolism and myocardial infarction. Second, the non-inferiority of tTXA relative to ivTXA in reducing total blood loss could not be drawn because of the limited study power. Third, the Hb drop of each patient in the present study is calculated as the decrease of Hb level plus the theoretical increase in Hb level caused by transfusion. In our hospital, one unit of PRBC contains 24 g hemoglobin on average. However, the exact reason for the higher Hb drop remains unclear, this study may not be generalized to centers with a lower. Fourth, the percentage of fracture cases was higher in the placebo group, which may have biased the results.

## Conclusion

IvTXA and tTXA reduce total blood loss by 327 ml and 252 ml compared with topical placebo, respectively, in patients undergoing unilateral primary THA. Contrary to expectations, this study cannot conclude the noninferiority of tTXA relative to ivTXA in reducing total blood loss in THA, indicating loss of the effect of tTXA has not been ruled out. However, the upper limit 95% CI of mean difference of total blood loss is only slightly higher than noninferiority margin and we found no significant difference in other efficacy outcomes between ivTXA and tTXA. In addition, basing on the results of the present study, we speculate that the intravenous route and topical route of TXA has similar efficacy for blood loss prevention. Finally, no significant difference was found between patients managed by TXA and patients managed by placebo for thromboembolic complication incidence.

## Supporting information

S1 Table The total blood loss formula. (PDF)

S2 Table CONSORT 2010 checklist of information to include when reporting a randomised trial. (PDF)

## References

[1] Saleh A, Small T, Chandran PA, Schiltz NK, Klika AK, Barsoum WK. Allogenic blood transfusion following total hip arthroplasty: results from the nationwide inpatient sample, 2000 to 2009. J Bone Joint Surg Am. 2014;96(18):e155 10.2106/JBJS.M.00825 25232085 PMC4159964

[2] Bierbaum BE, Callaghan JJ, Galante JO, Rubash HE, Tooms RE, Welch RB. An analysis of blood management in patients having a total hip or knee arthroplasty. J Bone Joint Surg Am. 1999;81(1): 2–10. 9973048 10.2106/00004623-199901000-00002

[3] Sehat KR, Evans R, Newman JH. How much blood is really lost in total knee arthroplasty? Correct blood loss management should take hidden loss into account. Knee. 2000;7(3): 151–155. 10927208 10.1016/s0968-0160(00)00047-8

[4] Lopez-Balderas N, Bravo E, Camara M, Hernandez-Romano P. Seroprevalence of hepatitis viruses and risk factors in blood donors of Veracruz, Mexico. J Infect Dev Ctries. 2015;9(3):274–282. 10.3855/jidc.4812 25771465

[5] Erstad BL. Systemic hemostatic medications for reducing surgical blood loss. Ann Pharmacother. 2001;35(7–8): 925–934. 10.1345/aph.10337 11485146

[6] Dunn CJ, Goa KL. Tranexamic acid: a review of its use in surgery and other indications. Drugs. 1999;57(6): 1005–1032. 10400410 10.2165/00003495-199957060-00017

[7] Yamasaki S, Masuhara K, Fuji T. Tranexamic acid reduces blood loss after cementless total hip arthroplasty? Prospective randomized study in 40 cases. Int Orthop. 2004;28(2):69–73. 10.1007/s00264-003-0511-4 15224162 PMC3474476

[8] Risberg B. The response of the fibrinolytic system in trauma. Acta Chir Scand Suppl. 1985;522:245–271. 3893000

[9] Benoni G, Lethagen S, Fredin H. The effect of tranexamic acid on local and plasma fibrinolysis during total knee arthroplasty. Thromb Res. 1997;85(3):195–206. 9058494 10.1016/s0049-3848(97)00004-2

[10] Jansen AJ, Andreica S, Claeys M, D’Haese J, Camu F, Jochmans K. Use of tranexamic acid for an effective blood conservation strategy after total knee arthroplasty. Br J Anaesth. 1999;83:596–601. 10673876 10.1093/bja/83.4.596

[11] Gill JB, Rosenstein A. The use of antifibrinolytic agents in total hip arthroplasty: a meta-analysis. J Arthroplasty. 2006;21(6):869–873. 10.1016/j.arth.2005.09.009 16950041

[12] Wong J, Abrishami A, El Beheiry H, Mahomed NN, Roderick Davey J, Gandhi R, et al Topical application of tranexamic acid reduces postoperative blood loss in total knee arthroplasty: a randomized, controlled trial. J Bone Joint Surg Am. 2010;92(15):2503–2513. 10.2106/JBJS.I.01518 21048170

[13] Alshryda S, Sukeik M, Sarda P, Blekinsopp J, Haddad FS, Mason JM. A systematic review and meta-analysis of the topical administration of tranexamic acid in total hip and knee replacement. Bone Joint J. 2014;96-B (8):1005–1015. 10.1302/0301-620X.96B8.33745 25086114

[14] Soni A, Saini R, Gulati A, Paul R, Bhatty S, Rajoli SR. Comparison between intravenous and intra-articular regimens of tranexamic acid in reducing blood loss during total knee arthroplasty. J Arthroplasty. 2014;29(8):1525–1527. 10.1016/j.arth.2014.03.039 24814890

[15] Patel JN, Spanyer JM, Smith LS, Huang J, Yakkanti MR, Malkani al. Comparison of intravenous versus topical tranexamic acid in total knee arthroplasty: a prospective randomized study. J Arthroplasty. 2014;29(8):1528–1531. 10.1016/j.arth.2014.03.011 24768543

[16] Sarzaeem MM, Razi M, Kazemian G, Moghaddam ME, Rasi AM, Karimi M. Comparing efficacy of three methods of tranexamic acid administration in reducing hemoglobin drop following total knee arthroplasty. J Arthroplasty. 2014;29(8):1521–1524. 10.1016/j.arth.2014.02.031 24726174

[17] Wei W, Wei B. Comparison of topical and intravenous tranexamic acid on blood loss and transfusion rates in total hip arthroplasty. J Arthroplasty. 2014;29(11):2113–2116. 10.1016/j.arth.2014.07.019 25155138

[18] Gomez-Barrena E, Ortega-Andreu M, Padilla-Eguiluz NG, Perez-Chrzanowska H, Figueredo-Zalve R. Topical intra-articular compared with intravenous tranexamic acid to reduce blood loss in primary total knee replacement: a double-blind, randomized, controlled, noninferiority clinical trial. J Bone Joint Surg Am. 2014;96(23):1937–1944. 10.2106/JBJS.N.00060 25471907

[19] Tuttle JR, Ritterman SA, Cassidy DB, Anazonwu WA, Froelich JA, Rubin LE. Cost benefit analysis of topical tranexamic acid in primary total hip and knee arthroplasty. J Arthroplasty. 2014;29(8):1512–1515. 10.1016/j.arth.2014.01.031 24630599

[20] Zhou KD, Wang HY, Yan YF, Hong WX, Feng JM. Clinical efficacy of tranexamic acid administration via different routes during total hip arthroplasty: study protocol for a prospective, paired, double-blind, randomized controlled clinical trial. Clin Trials Orthop Disord. 2016;1:1–7. Available from: http://www.clinicalto.com/text.asp?2016/1/1/1/178819

[21] Martin JG, Cassatt KB, Kincaid-Cinnamon KA, Westendorf DS, Garton AS, Lemke JH. Topical administration of tranexamic acid in primary total hip and total knee arthroplasty. J Arthroplasty. 2014;29(5):889–894. 10.1016/j.arth.2013.10.005 24238825

[22] Alshryda S, Mason J, Sarda P, Nargol A, Cooke N, Ahmad H et al Topical (intra-articular) tranexamic acid reduces blood loss and transfusion rates following total hip replacement. J Bone Joint Surg Am. 2013;95(21):1969 24196467 10.2106/JBJS.L.00908

[23] Nadler SB, Hidalgo JH, Bloch T. Prediction of blood volume in normal human adults. Surgery. 1962;51(2):224–32. 21936146

[24] Gross JB. Estimating allowable blood loss: corrected for dilution. Anesthesiology. 1983;58(3):277–280. 6829965 10.1097/00000542-198303000-00016

[25] Zufferey P, Merquiol F, Laporte S, Decousus H, Mismetti P, Auboyer C et al Do antifibrinolytics reduce allogeneic blood transfusion in orthopedic surgery? Anesthesiology. 2006;105(5):1034–46. 17065899 10.1097/00000542-200611000-00026

[26] Kagoma YK, Crowther MA, Douketis J, Bhandari M, Eikelboom J, Lim W. Use of antifibrinolytic therapy to reduce transfusion in patients undergoing orthopedic surgery: a systematic review of randomized trials. Thromb Res. 2009;123(5):687–96. 10.1016/j.thromres.2008.09.015 19007970

[27] Ho KM, Ismail H. Use of intravenous tranexamic acid to reduce allogeneic blood transfusion in total hip and knee arthroplasty: a meta-analysis. Anaesth Intensive Care. 2003;31(5):529–37. 14601276 10.1177/0310057X0303100507

[28] Nilsson IM. Clinical pharmacology of aminocaproic and tranexamic acids. J Clin Pathol Suppl (R Coll Pathol). 1980;14:41–7. 7000846 PMC1347104

[29] Suenson E, Lutzen O, Thorsen S. Initial plasmin-degradation of fibrin as the basis of a positive feed-back mechanism in fibrinolysis. Eur J Biochem. 1984;140(3):513–22. 6233145 10.1111/j.1432-1033.1984.tb08132.x

[30] Benoni G, Lethagen S, Nilsson P, Fredin H. Tranexamic acid, given at the end of the operation, does not reduce postoperative blood loss in hip arthroplasty. Acta Orthop Scand. 2000;71(3):250–4. 10.1080/000164700317411834 10919295

[31] Wei Z, Liu M. The effectiveness and safety of tranexamic acid in total hip or knee arthroplasty: a meta-analysis of 2720 cases. Transfus Med. 2015;25(3):151–62. 10.1111/tme.12212 26033447

[32] Wind TC, Barfield WR, Moskal JT. The effect of tranexamic acid on transfusion rate in primary total hip arthroplasty. J Arthroplasty. 2014;29(2):387–389. 10.1016/j.arth.2013.05.026 23790499

[33] Oremus K, Sostaric S, Trkulja V, Haspl M. Influence of tranexamic acid on postoperative autologous blood retransfusion in primary total hip and knee arthroplasty: a randomized controlled trial. Transfusion. 2014;54(1):31–41. 10.1111/trf.12224 23614539

[34] North WT, Mehran N, Davis JJ, Silverton CD, Weir RM, Laker MW. Topical vs intravenous tranexamic acid in primary total hip arthroplasty: a double-blind, randomized controlled trial. J Arthroplasty. 2016;31(5):1022–1026. 10.1016/j.arth.2015.11.003 26703193

[35] Konig G, Hamlin BR, Waters JH. Topical tranexamic acid reduces blood loss and transfusion rates in total hip and total knee arthroplasty. J Arthroplasty. 2013; 28(9):1473–1476. 10.1016/j.arth.2013.06.011 23886406 PMC3807723

[36] Yi Z, Bin S, Jing Y, Zongke Z, Pengde K, Fuxing P. Tranexamic acid administration in primary total hip arthroplasty: a randomized controlled trial of intravenous combined with topical versus single-dose intravenous administration. J Bone Joint Surg Am. 2016;98(12): 983–991. 10.2106/JBJS.15.00638 27307358

[37] Sun Y, Jiang C, Li Q. A systematic review and meta-analysis comparing combined intravenous and topical tranexamic acid with intravenous administration alone in THA. PLOS ONE. 2017;12(10): e186174 10.1371/journal.pone.0186174 29016673 PMC5634626

