# Supplementary material for: Is topical or intravenous tranexamic acid preferred in total hip arthroplasty? A randomized, controlled, noninferiority clinical trial
Source: PLoS One. 2018 Oct 2;13(10):e0204551. doi: 10.1371/journal.pone.0204551 (PMC6168126; doi:10.1371/journal.pone.0204551)
Supplement: S1 Table — (PDF) [file pone.0204551.s001.pdf]

Blood loss formula

TBL = BV * (Hct_{pre}–Hct_{post} /Hct_{ave})

BV = k1 * height + k2 * weight ^{2} + k3;

k1 = 0.3669, k2 = 0.03219, and k3 = 0.6041 for men; and k1 =0.3561, k2 = 0.03308, and k3 = 0.1833 for women.

Hct_{pre} = the preoperative Hct level.

Hct_{post} = the minimum postoperative Hct level. （we use postoperative D3 hct cause it is lower than that in D1 and D5 ）

Hct_{ave} = the average of the Hct_{pre} and Hct_{post}.

BV = the patient’s blood volume (BV, ml)
